# A third species of *Polyspatha*, an Africanendemic genus of Commelinaceae

**DOI:** 10.3897/phytokeys.3.1181

**Published:** 2011-05-30

**Authors:** Robert B. Faden

**Affiliations:** Department of Botany, National Museum of Natural History, Smithsonian Institution, MRC 166, PO Box 37012, Washington, DC 20013–7012, U.S.A.

**Keywords:** Cameroon, Dahomey gap, seed morphology, Uganda, Ivory Coast, morning anthesis, disjunct distribution

## Abstract

*Polyspatha oligospatha* Faden, the third species in a small African endemic genus of Commelinaceae, is described. It is widespread but has been overlooked because of its small stature and resemblance to small plants of *Polyspatha paniculata*. It differs from both *Polyspatha paniculata* and *Polyspatha hirsuta*, the two other species, by its leaf pubescence, fewer, more widely spaced and usually patent spathes, deeply ridged seeds with numerous knobby, transversely interrupted ridges, and morning anthesis. It occurs throughout the Congolian forests from Cameroon to Uganda, but it is also disjunct in Ivory Coast, across the Dahomey gap.

## Introduction

The African endemic genus *Polyspatha* (Commelinaceae) has generally been considered to consist of two species (Morton 1967; Brenan 1968). It was described by Bentham (1849) for a single West African species, *Polyspatha paniculata* Benth. In his monograph of the family, Clarke (1881) also recognized *Polyspatha paniculata* var. *glaucescens* C. B. Clarke, which he subsequently abandoned in the Flora of Tropical Africa (Clarke 1901). Clarke’s variety was raised to species rank as *Polyspatha glaucescens* (C. B. Clarke) Hutch. in the first edition of the Flora of West Tropical Africa (Hutchinson 1936) but the name was abandoned by Morton in his survey of Commelinaceae of West Africa (Morton 1967) and treated in synonymy under *Polyspatha paniculata* in the second edition of the Flora of West Tropical Africa (Brenan 1968). The second currently recognized species of *Polyspatha*, *Polyspatha hirsuta* Mildbr., was described by Mildbraed (1925). Faden (1998) recorded *Polyspatha* as having three species, but the third species had not been described.

When I first began working on the Commelinaceae of Cameroon and examined the *Polyspatha* specimens at the Herbier National du Cameroun (YA) in Yaoundé, Cameroon in 1986, it appeared that there were three distinct species of *Polyspatha*, not the usually accepted two. I came to the same conclusion from studies at the Muséum National d’Histoire Naturelle, Paris (P) the following year. Later studies at the Royal Botanic Gardens Kew (K), in connection with work on the Flora of Tropical East Africa, confirmed that the same three species also occurred in Uganda, some 2000 km away. Studies of collections from other herbaria, particularly the National Botanic Garden of Belgium, Brussels (BR), confirmed that the three species also were present in the intervening territory. It was particularly significant that this species was also found to occur in Ivory Coast, almost 1900 km from the nearest locality in Cameroon.

## Methods

More than 540 herbarium specimens of *Polyspatha* were studied from the following institutions: all the collections from BM, BR, EA, K, NY, MO, US, WAG and YA, and some of the collections from P. Living material of *Polyspatha oligospatha* (*Poulsen 1275*, originally from Uganda, and *Faden et al. 86/2*, originally from Cameroon) was cultivated at the Smithsonian Institution Botany Research Greenhouse, in Suitland, Maryland, USA and provided data on flowering times, root tips for chromosome counts, seed collections and liquid preserved flowers. A mitotic chromosome count was obtained using the techniques of Faden and Suda (1980). The seeds were photographed using an Olympus SZX-12MDU dissecting microscope, equipped with an Olympus Q-Color 5 camera, using Image-Pro and extended depth of field (EDF). The distribution map was created using ArcGIS once latitudes and longitudes were determined or estimated for the collections seen.

## Results

### Polyspatha oligospatha

Faden sp. nov.

urn:lsid:ipni.org:names:77111569-1

http://species-id.net/wiki/Polyspatha_oligospatha

#### Latin

Polyspatha oligospatha *Faden sp. nov. a* Polyspatha paniculata *foliis trichomatibus uniseriatis in pagina superiore instructis, spathis plerumque minus numerosis differt; a* Polyspatha hirsuta *pubesentia foliorum plerumque multo sparsa trichomatibus uniseriatis in pagina inferiore semper carentibus, spathis minus congestis minus numerosis minus pubescentibus et plerumque patentibus differt; et ab ambo ordinatione testae et anthesi mane differt. Type: Uganda, Zintengese [=Zintengeze], (Mabira), September 1922, R. A. Dummer 5531 (US!, holo, K!, iso).*

#### Description.

**Figure 1.**
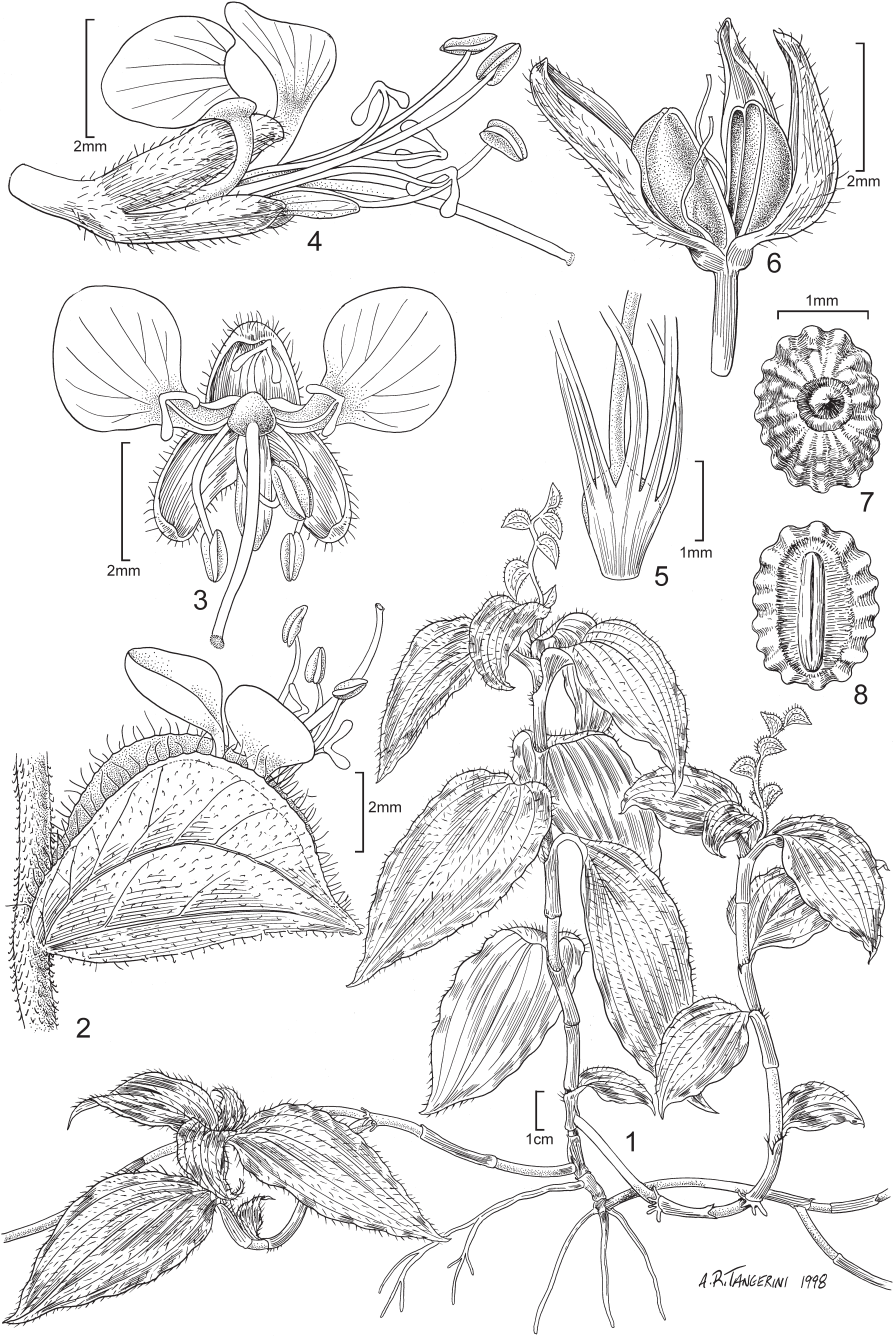
*Polyspatha oligospatha* Faden, sp. nov. 1. Habit. 2. Spathe with open flower, side view. 3. Flower, front view. 4. Flower, side view. 5. Stamen and staminode filaments, showing basal fusion. 6. Dehisced capsule. 7. Seed, dorsal view. 8. Seed, ventral. All from *Poulsen 1275* (originally from Uganda; cultivated at the Smithsonian Institution). Illustration by A. R. Tangerini.

**Plate 1.**
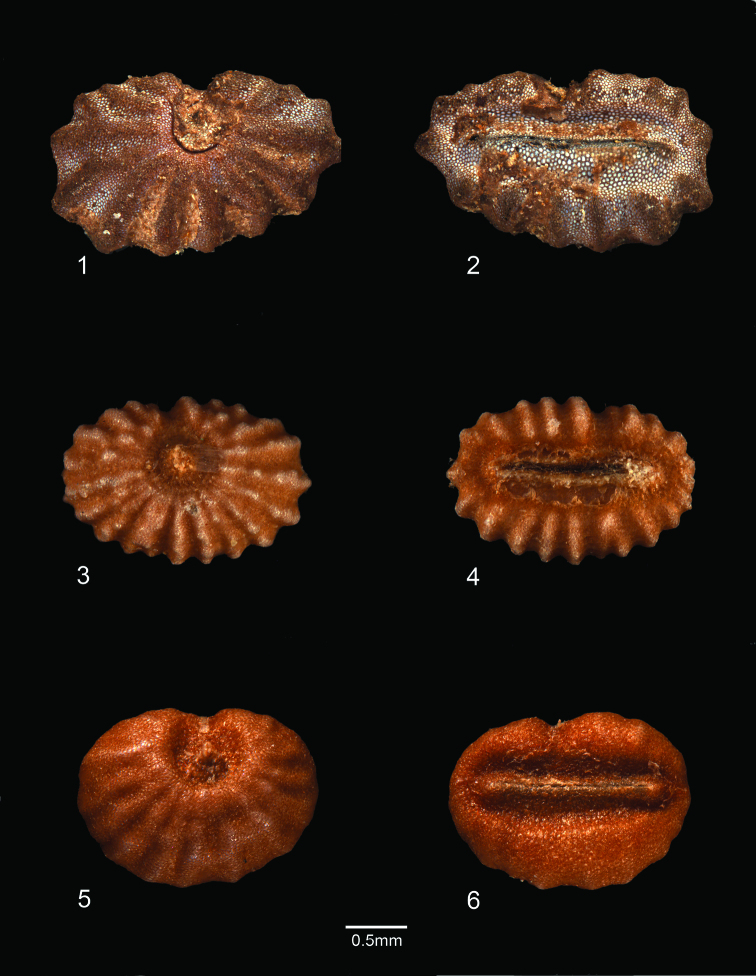
Seeds of *Polyspatha* species **1–2** *Polyspatha paniculata* Benth. **1** Seed, dorsal view **2** Seed, ventral view. (From *Faden et al. 74/35* from Ghana, cultivated at Smithsonian Institution) **3–4** *Polyspatha oligospatha* Faden, sp. nov. **3** Seed, dorsal view. 4. Seed, ventral view. (From *de Wilde & de Wilde-Duyfjes 2647* [BR] from Cameroon) **5–6** *Polyspatha hirsuta* Mildbr. **5** Seed, dorsal view **6** Seed, ventral view. (From *Kahn 92-1* [US] from Cameroon).

Stoloniferous perennial with erect shoots 5–20(-30) cm tall; internodes puberulous with hook-hairs, very rarely also with short, uniseriate hairs. Leaves usually subclustered terminally on the flowering shoot, sheaths 0.8–1.5 cm long, puberulous with hook-hairs (Tomlinson 1966), very rarely also with short, uniseriate hairs, ciliate at the apex with hairs to 3 mm long; lamina petiolate, elliptic or broadly elliptic to ovate or ovate-orbicular, rarely oblong-elliptic, 3–9(-11) × 2–4.5 cm, apex acute to acuminate (to abruptly acute or mucronate), base cuneate to broadly cuneate, margins planar to slightly undulate, scabrous, adaxial surface with scattered, patent, long, uniseriate hairs, abaxial puberulous with hook-hairs. Inflorescence a terminal, simple or compound thyrse 2–7 cm long, up to 7.5 cm wide, the compound thyrses composed of up to 4, closely associated, erect to patent or declinate, terminal and axillary (from the upper leaves) simple thyrses, none of the axillary thyrses perforating a leaf sheath, each simple thyrse consisting of a short peduncle and an elongate, zigzag, retrosely puberulous (with hook-hairs) axis, to which are attached 4–8 distichous spathes; spathes at first erect, then patent, rarely becoming deflexed against the thyrse axis, attached 3–11 mm apart, usually not overlapping the one below (on the opposite side) (occasionally slightly overlapping it), 6.5–10(-12) mm long, (3-)4–7(-9) mm wide (folded), apex acute to rounded, sometimes mucronate, or sometimes the proximal spathes acuminate, surfaces brown or brownish at maturity, at least along the midrib (folded edge), prominent longitudinal veins usually absent, surface cells (in the brown parts) lustrous, distinctly bead-like at 20× or higher magnification, surfaces puberulous with hook-hairs, sometimes with short, uniseriate hairs along the midrib area, margins ciliolate with hairs usually all <1.5 mm long; cincinni ca. 2–3-flowered; bracteoles ovate, with a few short hairs on the margins. Flowers bisexual, 3.5–8 mm wide; pedicels 1–2.5 mm long, glabrous or sparsely puberulous; sepals free, lanceolate or lanceolate-oblong to ovate, 3–4.5 × 1–2.3 mm, puberulous with hook-hairs and short, uniseriate hairs, white or hyaline white, outer sepal hooded distally; corolla white, upper petals clawed, 5–7 × 2.5–3 mm, limb ovate, 2–3 × 2.5–3 mm, claw 3.5–5 mm long, lower petal linear to oblong, (2-)3–4.7 × (0.2-)0.8–1 mm; filaments of stamens and staminodes fused basally, glabrous, white; staminodes 3, posterior, equal, filaments 3–6 mm long, antherodes pointing forwards, V-shaped, yellow, the lobes divergent, oblanceolate, 0.6–0.8 mm long; fertile stamens 3, anterior, filaments subequal, but the medial slightly shorter than the laterals, filaments 4.5–8.5 mm long, anthers ovate-elliptic to elliptic, 1–1.7 mm long, yellow, dehiscence extrorse, pollen yellow; ovary sessile, obovoid, dorsiventrally flattened, ca. 1 mm long and wide, style exceeding the stamens, 5.5–8.5 mm long, white, stigma capitate, white. Capsules bilocular, bivalved, 2-seeded, broadly elliptic, 2.5–3 × 2.5–3 mm, sometimes constricted between the seeds, tan, apex emarginate, cells of the outer capsule wall ±isodiametric. Seeds elliptic in outline, 1.6–2.2(-2.5) × 1.3–1.5(-1.6) mm, testa radiately ribbed with (17-)18–23 prominent ribs, the ribs more or less knobby and transversely interrupted, surface tan, sometimes more or less exposed except for some darker brown, matted material between the ribs, sometimes mainly covered by this material except for the rib tops; embryotega semidorsal; hilum straight to slightly curved, 1/2 to 2/3 the length of the seed (Figure 1; Plate 1: 3, 4)

#### Distribution.

**Figure 2.**
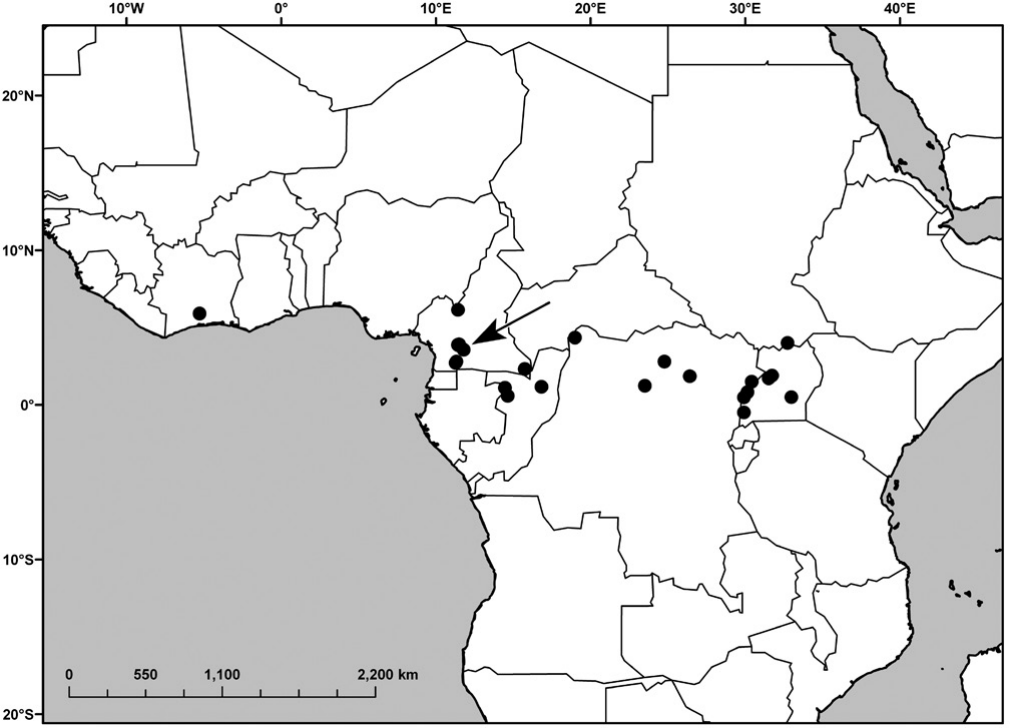
Distribution of *Polyspatha oligospatha* Faden, sp. nov. The arrow indicates the location of the collection *Letouzey 11465*, which is atypcial for the species (see text).

Ivory Coast, Cameroon, Republic of Congo (Congo-Brazzaville), Democratic Republic of Congo (Congo-Kinshasa), Sudan, Uganda (Figure 2).

#### Habitat.

Understory in evergreen and semi-deciduous forests, forest relicts and other moist, shaded habitats in savanna, shaded cultivation, especially of cacao; ca. (15-)600–1220 m.

#### Chromosome number.

2*n* = 28 (from *Faden et al. 86/2* from Cameroon, cultivated at the Smithsonian Institution [Faden unpublished]).

#### Phenology and anthesis.

A total of 24 flowering collections of *Polyspatha oligospatha* have been seen, with specimens collected from all months except April and May. Anthesis occurs in the morning, based on the following records. *Poulsen 1275* from Uganda, cultivated at the Smithsonian Institution Botany Research Greenhouse in 1997, had flowers open by 9:30 a.m. and fading at noon. Two collections from Cameroon, *Hall & Kahn 075/93* and *Keating 90–13* (both US), record this species as flowering in the morning and fading about noon.

*Polyspatha oligospatha* is easily recognizable but also readily overlooked, judging by its wide distribution and modest number of collections. It is distinctive because of its small stature, leaves with long, uniseriate hairs only on the adaxial surface, small inflorescences with small, well-spaced, often patent (i.e. not becoming deflexed) spathes whose surface is composed entirely or partly of lustrous, brown, bead-like cells, and seeds with numerous, radiating, knobby ridges that are transversely interrupted (Figure 1: 7, 8; Plate 1: 3, 4).

#### Specimens seen.

#### Cameroon:

Adamoua Region: Near Kongi (10 km NW of Kimi-Bankim on Foumban - Banyo route), 27 June 1967, *Letouzey 8742* (K, P, WAG) [two additional sheets of this collection at P are *Polyspatha paniculata*]. Center Region: N’Kolbisson, ca. 8 km W of Yaoundé, 2 Dec. 1963, *de Wilde & de Wilde-Duyfjes 1369* (P, WAG); same locality, 7 June 1964, *de Wilde & de Wilde-Duyfjes* *2647* (BR, K, WAG, YA); Nkolbisson, 8 km W of Yaoundé, Akouandoué Hill S of the town, 23 Jan.1986, *Faden, Satabié & Mpom 86/2* (K, P, US, YA); Nkolbisson, 8 km W of Yaoundé, 3 km NW of the town, 24 Jan. 1986, *Faden, Satabié & Mpom 86/22* (US); Mt. Febe, Yaoundé, 4 July1987, *Manning 2166* (K, MO, WAG); Colline au N de Nkolbison (8 km W Yaoundé), 6 Feb.1963, *Raynal & Raynal 9445* (P, YA). East Region: Boumba-Ngoko Dept., Moloundou Arrondissement, Lobeke swamp/savanna, adjacent to camp (site of an old sport hunting camp), 6 Feb.1993, *Hall & Kahn 075/93* (K, P, US, YA, WAG). South Region: Ntem Departement, Ako’okas, 40 km SE of Ebolowa (by road), 30 June 1993, *Keating 90–13* (US); same locality, 4 Aug. 1993, *Keating 90–13 bis* (K, P, US, YA); Rocheur de Mokomessi, 20 km NNW of Zoetélé, 35 km NNW of Sangmelima, 9 July1992, *Letouzey 11465* (BR, K, P, YA); Ebolowa - Si I (W d’Ebolowa), basses pentes d’une colline dominant la ville, 9 Mar. 1963, *Raynal & Raynal 10340* (P, YA).

#### Congo Republic:

Bassin de l’Alimo-Likoula, Réserve de Chasse de M’Boko, salime au bord de la Lekoli près du campement, 10 Aug. 1961, *Descoings 9059* (P)**;** Grand escarpement d’Odzala, au Nord de la cascade dorée, 27 Nov. 1996, *Lejoly 96/961* (BR); Inter Bonga et Wesso (Sanga), Aug. 1899, *Schlechter 12714* (BR) [mixture with *Polyspatha paniculata*; K sheet of this number is pure *Polyspatha paniculata*].

#### Democratic Republic of Congo:

Forestier Central (VI):en aval de Barumbu, 27 Oct. 1913, *Bequaert 971* (BR); Panga, 19 Dec. 1913, *Bequaert 1561* (BR); Uelé, Route Buta – Buna, 15 Oct. 1905, *Seret 70* (BR) [2 sheets at BR, each with one plant of *Polyspatha oligospatha* (renumbered *Seret 70A*) and one of *Polyspatha paniculata* (renumbered *Seret 70B*); mapped as “Buta”)]. Ubangi-Uele (VII): Zongo (Ubangi), Nov. 1930, *Lebrun 1729* (BR). Lacs Edouard et Kivu (IX): Vallée de la Semliki, a l’est de Beni, July 1929, *Humbert 8796* (BR, P).

#### Ivory Coast:

Divo Forest, Oct. 1959, *Aké Assi 5707* (K); North Divo Forest Reserve, 12 Oct. 1959, *Fosberg 40559* (K, US).

#### Sudan:

Equatoria Province, Torit District: Tallanga Forest, 28 Dec. 1949, *Jackson 1005* (BM).

#### Uganda:

Bunyoro (Masindi) District: Pabidi Forest, 14 Sep. 1995, *Poulsen et al. 958* (C, US); Budongo Forest Reserve, Kanyo-Pabidi Block, 4 Feb. 1996, *Poulsen et al.* 1180 (C); Budongo Forest Reserve, between the Royal Mile and the Nature Reserve, close to the border of the Nature Reserve, at the campsite at the Nyabisabo River (gap), 4 Dec.1996, *Poulsen et al. 1245* (US) & *1246* (K, MO); Budongo Forest, 17 July 1969, *Stewart in EAH14170* (EA, K). Kigezi District: Just outside Queen Elizabeth Nat. Park, South Maramagambo Central Forest Reserve, 6.4–11.2 km up Kaizi-Bitereko road which is off the Katunguru-Ishsha road (Congo road), 18 Sep.1969, *Faden et al. 69/1113* (BR, EA); South Maramagambo Central Forest Reserve, just outside Queen Elizabeth Nat. Park, 8 June 1969, *Lock 69/155* (EA); Maramagambo Forest, 18 Sep.1969, Lye et al. 4117 (K). Mengo District: Zintengese [=Zintengeze], (Mabira), Sep. 1922, *Dummer 5531* (K, US). Toro District: “Semliki District,” 31 Oct. 1905, *Daws 683* (K); Bwamba County, 1.2 km S of Sempaya, 23 Sep. 1969, *Faden, Evans & Lye 69/1256* (EA); Bwamba, Buyayu-Sempayo road, Oct. 1929, *Liebenberg 922* & *922A* (K) [both are mixtures with *Polyspatha paniculata*].

#### The three species of Polyspathia may be separated by the following key:

**Table:** 

1a	Lamina lacking long, uniseriate hairs, often scabrous above; seeds ribbed, with (12-)14–18 smooth, uninterrupted ribs	*Polyspathia paniculata*
1b	Lamina with long, uniseriate hairs, at least on the adaxial surface, never scabrous above; seeds either shallowly ribbed-reticulate or deeply ribbed with (17-)18–23 prominent, knobby ribs that are transversely interrupted	2
2a	Long, uniseriate hairs present on the adaxial leaf surface and summits of the leaf sheaths, always lacking from the abaxial leaf surface and usually elsewhere; spathes well spaced, usually patent to slightly deflexed; cells of the spathe surface, at least near the midrib, but often all over, lustrous, brown, and bead-like under a 20× lens; seeds deeply ribbed, the ribs knobby and transversely interrupted	*Polyspathia oligospatha*
2b	Long, uniseriate hairs usually present on both leaf surfaces, the internodes and sheath surfaces (lacking only in some collections from Nigeria and further west); spathes crowded, becoming deflexed against the inflorescence axis; cells of the spathe surface dull, neither brown nor bead-like under a 20× lens; seeds shallowly ribbed-reticulate, the ribs neither knobby nor transversely interrupted	*Polyspathia hirsuta*

## Discussion

*Polyspatha oligospatha* [etymology: many spathes, few spathes] differs from *Polyspatha paniculata* by the consistent presence of long, uniseriate hairs on the adaxial leaf surface (vs. none), which is never scabrous (vs. often scabrous) and by the usually fewer, smaller, blunter, more widely spaced and less deflexed spathes. It differs from *Polyspatha hirsuta* by the usually sparser and shorter uniseriate hairs on the adaxial leaf surface, the absence of such hairs from the abaxial leaf surface and usually the internodes and sheaths (except for the sheath summit), and the inflorescence consisting of fewer, less congested spathes with shorter marginal hairs and with lustrous, brown, bead-like cells on the entire spathe surface or mainly near the midrib (vs. such cells completely lacking). The new species differs from both other species by its generally smaller stature (but see below) and seeds with numerous, knobby, transversely interrupted ridges as compared with generally fewer, smoother, uninterrupted ridges in the seeds of *Polyspatha paniculata* and testa shallowly ridged-reticulate in *Polyspatha hirsuta* (Figure 1: 7, 8; Plate 1).

Leaf pubescence in *Polyspatha hirsuta* can be quite variable, but plants with sparse pubescence or with long hairs confined to the adaxial leaf surface are restricted to Nigeria and further west, overlapping with *Polyspatha oligospatha* only in Ivory Coast. Thus throughout nearly all of the range of *Polyspatha oligospatha*, the pubescence differences of the leaf lamina, sheath, and internodes between *Polyspatha oligospatha* and *Polyspatha hirsuta* are consistent. The often much less pubescent plants of West African *Polyspatha hirsuta* still exhibit the stature, leaf shape and spathes that are typical of the species, so they would not be readily confused with *Polyspatha oligospatha* even if the two species were more broadly sympatric.

The new species has been confused in the past with small specimens of *Polyspatha paniculata* which can be of similar stature and appearance. Some may even have very similar looking spathes and smaller capsules and seeds than in typical plants of *Polyspatha paniculata*. Such plants can be separated from *Polyspatha oligospatha* by the absence of long, uniseriate hairs from the leaves, the spathes generally more crowded, overlapping and deflexed against the inflorescence axis, and the seeds with generally fewer, smooth, uninterrupted ridges, as in typical *Polyspatha paniculata* (Plate 1–1).

This species has been overlooked or misinterpreted partly because of Clarke’s *Polyspatha paniculata* var. *glaucescens*, which was raised to a species in the first edition of Flora of West Tropical Africa (FWTA) by Hutchinson (1936), although it had already been abandoned by Clarke (1901) in Floral of Tropical Africa. From a study of the type of this variety, Brenan (1968) in the second edtion of FWTA correctly concluded that it was no more than a depauperate form of *Polyspatha paniculata*, agreeing with Morton (1967). This led Brenan to annotate the only specimen at Kew of *Polyspatha oligospatha* from West Tropical Africa, *Aké Assi 5707* from Ivory Coast, as “small form--I do not consider *Polyspatha glaucescens*...as distinct.” On the other hand, it is clear that Brenan had begun to suspect that there was more variation in the genus than the two West African species by his note on the species folder of *Polyspatha paniculata* from Uganda, which included specimens of *Polyspatha oligospatha*: “Some of these Uganda sheets look distinct and should be investigated.”

The collection *Letouzey 11465* (Figure 2, arrow) from Cameroon is accepted as *Polyspatha oligospatha* with reservation. The pubescence of the upper leaf surface is more like that of *Polyspatha hirsuta*, with the uniseriate hairs longer, denser and paler than in typical *Polyspatha oligospatha*. However the absence of uniseriate hairs from the lower leaf surface, sheaths (except for the summits) and internodes distinguishes it from all Cameroonian specimens of *Polyspatha hirsuta*. Moreover, the outer surface of the small spathes is composed solely of lustrous, dark brown, bead-like cells, which are present in all specimens of *Polyspatha oligospatha* but none of *Polyspatha hirsuta*. The short marginal hairs on the spathes also agree with *Polyspatha oligospatha*, but are unusual for *Polyspatha hirsuta* in the same region. Overall, *Letouzey 11465* is best treated as an atypical specimen of *Polyspatha oligospatha*.

Three sheets of *Letouzey 11465* have been seen but those at P and YA were not critically examined. Thus my observations and conclusions have been based solely on the duplicate of this collection from BR. Unfortunately, it lacks seeds, which would be diagnostic.

*Manning 2166* is unusual for *Polyspatha oligospatha* in several characters. The internodes and surface of the leaf sheaths bear numerous uniseriate hairs, a pubescence that has not been noted in any other collection of *Polyspatha oligospatha*. The spathes are also more pubescent than usual, bearing many, long, uniseriate hairs on the surface as well as along the midrib. The longest marginal hairs are >1.5 mm long, the normal maximum for *Polyspatha oligospatha*. Moreover, the spathes have very few lustrous, brown, bead-like cells and these are confined to the midrib area towards the spathe bases. In all these characters *Manning 2166* resembles *Polyspatha hirsuta*. It differs from that species by its shorter stature, absence of long hairs from the abaxial leaf surface--they are typically present in Cameroonian collections--shorter uniseriate hairs on the internodes and sheath surfaces, and more spaced, patent and pointed spathes that bear at least a small number lustrous, brown, bead-like cells along the midrib. *Manning 2166* might possibly represent a hybrid of *Polyspatha oligospatha* and *Polyspatha hirsuta*, but in view of the different times of anthesis in these species, I think it is best considered an atypical specimen of *Polyspatha oligospatha*. As in the case of *Letouzey 11465*, only the WAG sheet of *Manning 2166*, of the three specimens seen, was studied in great detail, thus reinterpretation might be necessary when the other sheets can be reexamined.

*Polyspatha oligospatha* has sometimes been collected as part of mixed collections with *Polyspatha paniculata*. I have seen five such mixed collections: *Lieberberg 922* & 922A(K) from Uganda, *Seret 70* (BR) from the Democratic Republic of Congo, *Schlechter 12714* (BR, K) from the Republic of Congo, and *Letouzey 8742* from Cameroon. Possibly when further duplicates of already seen collections are examined in additional institutions, other mixed collections will be discovered. Thus far no mixed collections have been seen of *Polyspatha oligospatha* and *Polyspatha hirsuta*.

The disjunct occurrence of *Polyspatha oligospatha* in Ivory Coast is probably not an artifact of under-collection because in both Ghana and Nigeria, the two larger of the four countries between Ivory Coast and Cameroon, there were collectors who took a great interest in Commelinaceae. J. K. Morton made numerous collections of Commelinaceae in Ghana and elsewhere and wrote several important papers on the family. D. P. Stanfield was an avid collector of Commelinaceae in Nigeria and was rewarded for his efforts and lengthy and detailed notes by having a new genus *Stanfieldiella* named for him by Brenan (1960). Thus although *Polyspatha oligospatha* is somewhat inconspicuous I would have expected it to have been collected in Ghana and/or Nigeria if it occurred there.

Another factor that makes this disjunct distribution interesting is that it places *Polyspatha oligospatha* on both sides of the Dahomey gap, the natural break in the rainforest that separates the Upper Guinean and Congolian forest blocs. A similar distribution pattern is shown by another Commelinaceae species, *Palisota ambigua* (P.Beauv.) C.B.Clarke, which Flora of West Tropical Africa (Brenan 1968) places in southern Nigeria but no further west. However, I observed four collections of this species from Ivory Coast at P in 1987, including *Jolly 39*, so it too spans the Dahomey gap. The question remains whether these Ivory Coast occurrences are relicts of a former, continuous distribution or represent more recent long-distance dispersal events. The berry fruits of the *Palisota* would obviously suggest the latter, but because *Polyspatha* seeds have no obvious means of dispersal—either short distance or long distance—it is more difficult to use the same argument for that disjunction.

*Polyspatha oligospatha* appears to be reproductively isolated from the other two species because of its morning anthesis and flowers fading about noon. In Flora of West Tropical Africa Brenan (1968) records the flowering times of *Polyspatha paniculata* as 2:30–5:00 pm and *Polyspatha hirsuta* as 2:30 until evening. Thus anthesis for *Polyspatha oligospatha* does not overlap with those of the other two species.

## Conclusion

*Polyspatha oligospatha* Faden, a new species described herein, is a widespread African rainforest species that has been overlooked but is readily distinguished from the other two species by its seed testa pattern, leaf pubescence, spathe characters and morning flowering.

## Supplementary Material

XML Treatment for Polyspatha oligospatha
